# Vector-borne pathogens in dogs from different regions of Afghanistan

**DOI:** 10.1186/s13071-026-07534-7

**Published:** 2026-07-08

**Authors:** Alireza Sazmand, Mariaelisa Carbonara, Zabihullah Fasehi, Giada Annoscia, Jairo Alfonso Mendoza-Roldan, Hassan Alimoradi, Ali Reza Mirzayee, Leili Moradi, Grazia Greco, Domenico Otranto

**Affiliations:** 1 Department of Pathobiology, Faculty of Veterinary Medicine, Bu-Ali Sina University, Hamedan, Iran; 2 Department of Veterinary Medicine, University of Bari Aldo Moro, Valenzano, Bari Italy; 3 Department of Paraclinic, Faculty of Veterinary Science, Kunduz University, Kunduz, Afghanistan; 4 Department of Preclinical Sciences, Faculty of Veterinary Science, Balkh University, Mazar-i-Sharif, Balkh Afghanistan; 5 Department of Veterinary Clinical Sciences, Jockey Club College of Veterinary Medicine and Life Sciences, City University of Hong Kong, Kowloon Tong, Hong Kong China; 6 Chulalongkorn University, Bangkok, Thailand

**Keywords:** Babesiosis, Bartonellosis, Domestic dogs, Hepatozoonosis, Leishmaniosis, One Health, Vector-borne pathogens, Zoonosis

## Abstract

**Background:**

Canine vector-borne pathogens (CVBPs) are increasingly relevant, particularly those of zoonotic concern, due to climate change and increased animal mobility. However, there is no information on the actual burden of these infections in canine populations in several Western and South-Central Asian countries, including Afghanistan. Therefore, this study aimed to investigate the molecular prevalence of CVBP infections in stray and owned dogs across regions of Afghanistan.

**Methods:**

From July 2020 to August 2024, a total of 100 dogs with outdoor lifestyles in four provinces of Afghanistan (i.e., Kabul, Kunduz, Mazar-i-Sharif, and Takhar) were blood-sampled and molecularly screened for *Hepatozoon* spp., *Babesia* spp., *Leishmania* spp., filarioid helminths, and *Bartonella* spp.

**Results:**

Overall, 81% of dogs tested positive for at least one VBP. *Hepatozoon canis* was the most common (68%; present in all provinces), followed by *Bartonella vinsonii* subsp. *berkhoffii* (18%; present in all provinces), *Babesia vogeli* (4%; present in 3 provinces), *Acanthocheilonema* sp. (4%; present in 3 provinces), *Leishmania infantum* (2%; in Kunduz), and *Babesia negevi* (1%; in Mazar-i-Sharif). Co-infections were detected in 12% of dogs.

**Conclusions:**

To the best of our knowledge, this is the first epidemiological study of CVBPs in Afghanistan, demonstrating the sympatric circulation of *H*. *canis*, *Bartonella vinsonii berkhoffii*, *L. infantum*, *B. vogeli*, *B. negevi*, and *Acanthocheilonema* sp. Overall, the findings underscore the importance of endoparasite and ectoparasite control in owned dogs, as well as the need to control feral animal populations to reduce VBP transmission.

**Graphical Abstract:**

**Figure:**
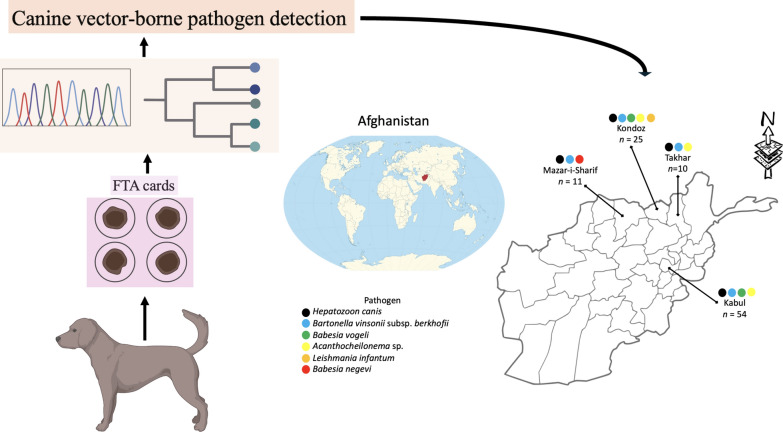

## Background

Canine vector-borne diseases (CVBDs), particularly those of zoonotic relevance, are attracting increasing attention due to multiple factors, including climate change and increased animal mobility [1]. The impact of these infections is even more pronounced in tropical and subtropical countries, where climatic conditions are optimal for the proliferation of competent arthropod vectors [2]. Additionally, in these regions, access to healthcare services in both human and veterinary medicine is limited, particularly for diagnostic and therapeutic facilities [3].

Among canine vector-borne pathogens (CVBPs), the genera *Bartonella*, *Leishmania*, *Hepatozoon*, *Babesia*, and filarial nematodes are notable for their zoonotic relevance [4]. In this context, *Bartonella* spp. are vector-borne hemotropic bacteria transmitted by fleas, lice, keds, sand flies, and biting flies [5], some of which can also infect humans. Specifically, at least seven zoonotic *Bartonella* species have been reported in dogs, namely *Bartonella vinsonii* subspecies *berkhoffii* (*Bvb*), *B. rochalimae*, *B. henselae*, *B. clarridgeiae*, *B. elizabethae*, *B. washoensis*, and *B. quintana* [5]. These species may cause a range of clinical manifestations, from subclinical to severe, potentially life-threatening disease [6]. Although several *Bartonella* species (e.g., *B. quintana*, *B. koehlerae*, *B. taylorii*, *B. elizabethae*, and *B. doshiae*) have been molecularly detected in rodent fleas from Kabul, Afghanistan [7], no data are available on canine bartonellosis in this area.

Domestic dogs are the primary reservoirs of *Leishmania infantum*, the causative agent of zoonotic visceral and cutaneous leishmaniosis in the Mediterranean basin, northern Africa, western Asia, and Brazil [8]. However, the prevalence of infection is often underestimated because diagnosing subclinical cases is challenging (e.g., the lack of highly specific serological tests), with over 80% of cases in some areas [9].

Dogs in the Asian continent were also reported to be infected with several zoonotic filarioids, including *Dirofilaria immitis*, which causes heartworm disease, and several agents of subcutaneous filariosis (i.e., *Dirofilaria repens*, *Dirofilaria asiatica,* and *Acanthocheilonema reconditum*) [10, 11], but the circulation of these agents in canine populations in Afghanistan has never been assessed.

In addition to the zoonotic parasites mentioned above, dogs worldwide can be infected with tick-borne apicomplexan protozoa, including *Hepatozoon canis* and *Babesia* spp. [12, 13]. Although hepatozoonosis is usually asymptomatic, severe infections can cause fever, lethargy, and anemia [14–16]. Conversely, canine babesiosis can be severe, characterized by anemia, jaundice, anorexia, and lethargy [17, 18].

Afghanistan, located in south-central Asia, has faced ongoing wars, political instability, and widespread poverty for nearly half a century [19]. These factors led to nomadic displacement and the abandonment of dogs, which later became strays in cities and villages. Furthermore, inadequate environmental management increased the number of stray dogs in urban areas, raising the risk of pathogen transmission to domestic animals and humans [19, 20]. Moreover, these circumstances pose substantial difficulties in sampling dogs, testing them for the most important CVBD-causing pathogens, and administering appropriate treatments [20].

Overall, data on CVBDs in Afghanistan are extremely limited, with scarce and outdated information on leishmaniasis and on the arthropod vectors that infest dogs [21, 22]. Therefore, this study aims to assess the molecular prevalence and distribution of VBDs in stray and owned dogs across four regions of Afghanistan.

## Methods

### Study areas and sample collection

**Fig. 1:**
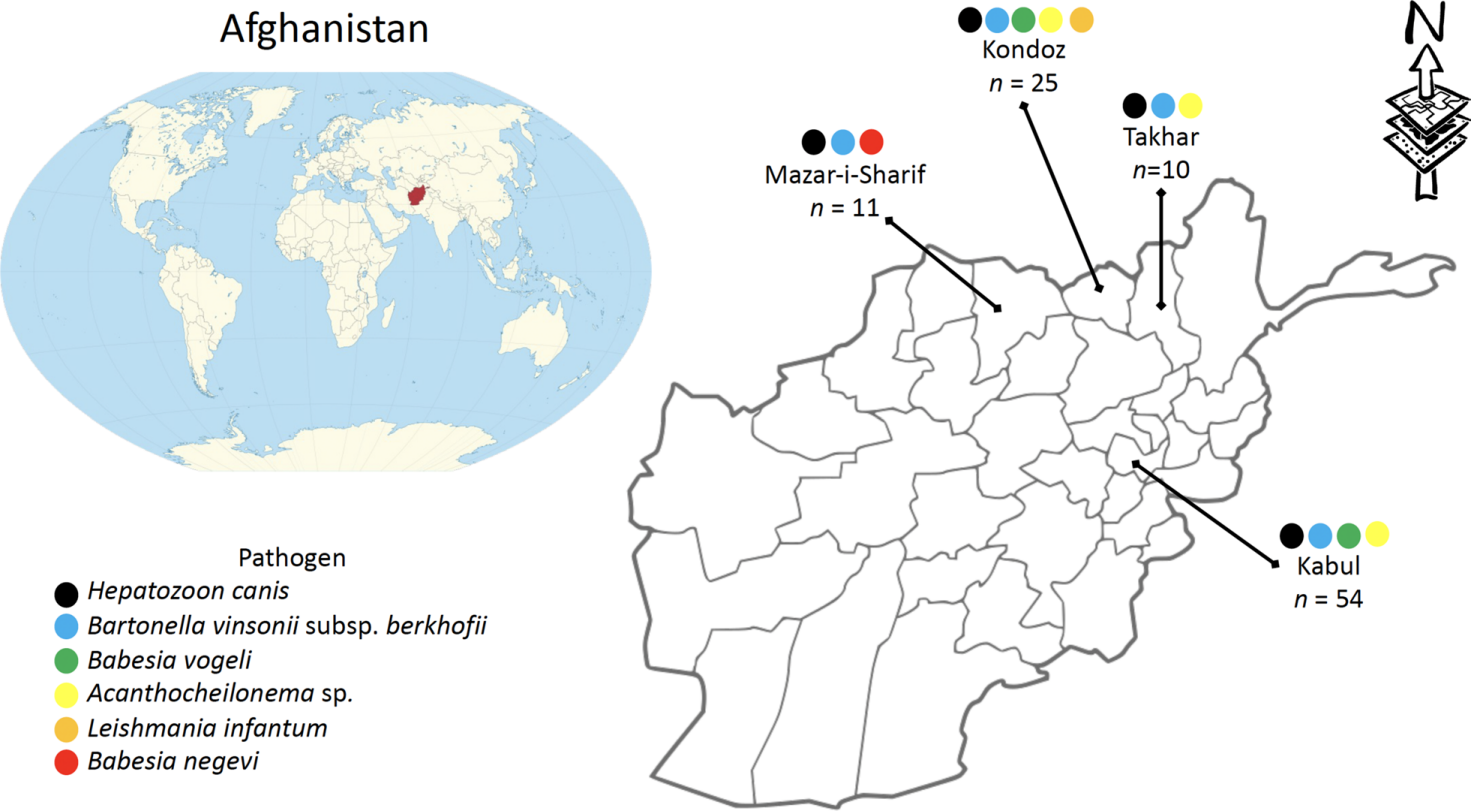
Geographical distribution of dogs’ blood samples and pathogens detected in this study

From July 2020 to August 2024, blood samples were collected from 100 dogs with outdoor access (i.e., all dogs stayed outside overnight), including both client-owned (*n* = 37) and stray (*n* = 63) dogs, across 4 provinces of Afghanistan (Fig. 1). At the time of sampling, animal data were recorded in individual files, including geographical origin, sex, age, and housing conditions. Dogs were grouped by age into < 1 year (G1), 1–3 years (G2), 3–5 years (G3), and > 5 years (G4). EDTA blood (approximately 100 μL) was spotted onto Whatman FTA® Elute sample collection cards (GE Healthcare, Little Chalfont, UK) and air-dried before shipment to the Unit of Parasitology, Department of Veterinary Medicine, University of Bari, Italy, for further molecular screening.

### Molecular analyses

Five 3.0-mm-diameter disks were punched from Whatman FTA cards (Uni-Core 150 punch; GE Healthcare, https://www.gelifesciences.com). Genomic DNA was extracted using the QIAamp® DNA Blood Mini Kit (Qiagen, Hilden, Germany) following the manufacturer’s instructions. Blood DNA samples were tested for *Babesia* spp., *Hepatozoon* spp., *Ehrlichia* spp./*Anaplasma* spp., *Bartonella* spp., and filarioids by conventional polymerase chain reaction (cPCR) and for *L. infantum* by real-time PCR (qPCR). *Leishmania* spp. qPCR-positive samples were further tested by cPCR for sequencing. All primer and probe details, along with PCR protocols, are reported in Table 1. Positive and negative controls were included in all thermocycling reactions.

**Table 1:** Primers and targeted pathogens investigated in this study

Pathogen	Target gene	Primers and probes (nucleotide sequence 5′–3′)	Method	Amplicon size (bp)	Reference
Piroplasmida*/Hepatozoon* spp.	18S rDNA	RLBF: GAGGTAGTGACAAGAAATAACAATA RLBR: TCTTCGATCCCCTAACTTTC	PCR	510	[55]
Filarioids	*cox*1	NTF: TGATTGGTGGTTTTGGTAA NTR: ATAAGTACGAGTATCAATATC	PCR	648	[56]
Filarioids	*12S rRNA*	Fila12SF: CGGGAGTAAAGTTTTGTTTAAACCG Fila12SR: CATTGACGGATGGTTTGTACCAC	PCR	330	[57]
Nematodes	ITS-2 rDNA	NC1: ACGTCTGGTTCAGGGTTGTT NC2: TTAGTTTCTTTTCCTCCGCT	PCR	390–485 for filarioids	[58]
*Ehrlichia* spp./*Anaplasma* spp.	*16S rRNA*	EHR16SD: GGTACCYACAGAAGAAGTCC EHR16SR: TAGCACTCATCGTTTACA GC	PCR	345	[59]
*Leishmania infantum*	kDNA minicircle	LEISH1: AACTTTTCTGGTCCTCCGGGTAG LEISH2: ACCCCCAGTTTCCCGCC Probe: TaqMan-MGB, FAM-AAAAATGGGTGCAGAAAT	qPCR	120	[60]
*Leishmania* spp.	ITS-1	L5.8S: 5′-TGATACCACTTATCGCACTT-3′ LITSR: 5′-CTGGATCATTTTCCGATG-3′	PCR	300	[61]
*Leishmania* spp.	*Hsp*70	F25: GGACGCCGGCACGATTKCT R617: CGAAGAAGTCCGATACGAGGGA	PCR	590	[62]
*Bartonella henselae*	*Pap31*	Pap31-BHSs: CCGCTGATCGCATTATGCCT Pap31-688as: AGCGATTTCTGCATCATCTGCT	PCR	526–557	[63]
*Bartonella vinsonii* subsp*. berkhoffii*	ITS	46-F: CCTCATTCTTTAAAAAAAGAGGGCTTTTTAAG 590-R: GAAAGCGCTAACCCCTAAACCGATT	PCR	697–710	[63]
*Bartonella* spp.	ITS	325 s: CTTCAGATGATGATCCCAAGCCTTYTGGCG 1100as: GAACCGACGACCCCCTGCTTGCAAAGCA	PCR	408–673	[63]

All cPCR products were run on a 2% agarose gel stained with GelRed (VWR International PBI, Milan, Italy) and visualized on a ChemiDoc Touch Gel Imaging System (Bio-Rad, CA, USA). Amplicons were purified enzymatically with Exonuclease I and FastAP Thermosensitive Alkaline Phosphatase, following the manufacturer’s protocol (Thermo Fisher Scientific, Waltham, MA, USA), and sequenced in both directions with the same primers used in PCR. Sequencing was performed with BigDye Terminator v3.1 chemistry on a 3130 Genetic Analyzer (Applied Biosystems, Foster City, CA, USA). Sequences were analyzed with BioEdit version 7.7 and compared with those in the GenBank database using the Basic Local Alignment Search Tool (BLAST; http://blast.ncbi.nlm.nih.gov/Blast.cgi) for species identification.

### Phylogenetic analysis and pairwise comparison

Sequences obtained in this study were aligned with homologous sequences from other organisms in GenBank using the ClustalW algorithm in MEGA12. The best-fitting nucleotide substitution model was determined before phylogenetic reconstruction using the “Find Best DNA Model (ML)” function in MEGA12. Model selection was based on the lowest Bayesian Information Criterion (BIC) score.

Phylogenetic relationships were inferred using the Maximum Likelihood (ML) method with dataset-specific models as follows: the Tamura model [23] was applied to the 18S rDNA and *cox*1 datasets; the Hasegawa–Kishino–Yano model [24] to the *12S* dataset; and the Kimura 2-parameter model to the ITS1 dataset [25]. Rate heterogeneity among sites was modeled with a discrete Gamma distribution (+ G) where appropriate, and a proportion of invariant sites (+ I) was included for selected datasets based on model selection results.

The robustness of the inferred tree topology was evaluated using 2000 bootstrap replicates (8000 for the *18S* dataset). All evolutionary analyses were performed in MEGA12 [26].

Pairwise comparisons of *cox*1 sequences were computed as the number of base substitutions per site using the Maximum Composite Likelihood model. The analysis included eight nucleotide sequences and considered all codon positions (1st, 2nd, and 3rd) as well as non-coding regions. Ambiguous positions were removed for each sequence pair using the pairwise deletion option, yielding a final dataset of 207 positions. All evolutionary analyses were conducted in MEGA version 12 [26], following the approach described previously [27].

### Statistical analysis

Prevalence analysis was performed using exact binomial 95% confidence intervals (CIs) for cPCR and qPCR results. Possible associations between VBP infection and animal data (i.e., province, sex, age, and living conditions) were assessed using chi-squared tests via an online tool (https://www.socscistatistics.com/tests/chisquare2/default2.aspx). A *P* value < 0.05 was considered statistically significant.

## Results

Overall, 81% of dogs (i.e., 81/100; CI 73.3–88.7%) tested positive for at least one VBP, with *H. canis* being the most common (68%; CI 58.9–77.1%), followed by *Bartonella* spp. (18%; CI 10.5–25.5%) and *Babesia* spp. (5%; CI 0.7–9.3%). Co-infection with two pathogens was observed in 12% of dogs (CI 5.6–18.4%) (Table 2, Fig. 1).

**Table 2:** Number and percentage of dogs (*n* = 100) PCR-positive for *Hepatozoon canis*, *Bartonella* spp., *Babesia* spp., *Acanthocheilonema* sp., and *Leishmania* spp

Pathogen	No. positive	95% CI
Single infection		
*Hepatozoon canis*	57	44–63
*Bartonella* spp.	8	3–12
*Leishmania* spp.	1	0–5
*Babesia* spp.	5	0.7–9.3
*Acanthocheilonema* sp.	2	0.5–7
Co-infection		
*Hepatozoon canis* + *Bartonella* spp.	9	4.9–17.1
*Leishmania* spp. + *Bartonella* spp.	1	0–3
*Hepatozoon canis* + *Acanthocheilonema* sp.	2	0–6.3

*Hepatozoon canis-*infected dogs (i.e., 100% nucleotide identity with canine isolates from Romania, KX712125-6) were found in all provinces investigated, with prevalence ranging from 63.6% to 80%, depending on the area considered (Table 3). Positivity was significantly higher in male dogs than in female dogs (*P* = 0.04) (Table 3). In total, 18 dogs (CI 10.5–25.5%) from all four provinces tested cPCR-positive for *Bartonella* spp., with prevalence varying by province (16–20%) (Table 3, Fig. 1). Specifically, 14 individuals were infected with *Bvb* and 4 with *Bartonella* spp.

**Table 3:** Risk factors and prevalence of canine vector-borne infections according to animal data

Variable	No	*Hepatozoon*			*Bartonella*			*Babesia*			*Acanthocheilonema*			*Leishmania*		
		Number (%)	95% CI^a^	χ2^b^ df^c^ *P*-value	Number (%)	95% CI^a^	χ2^b^ df^c^ *P*-value	Number (%)	95% CI^a^	χ2^b^ df^c^ *P*-value	Number (%)	95% CI^a^	χ2^b^ df^c^ *P*-value	Number (%)	95% CI^a^	χ2^b^ df^c^ *P*-value
Geographical origin (city)																
Kabul	54	35 (64.8)	52.1–77.6	χ2 = 1.1936 df = 3 *P* = 0.754536	10 (19)	8.2–28.9	χ2 = 0.1049 df = 3 *P* = 0.991239	2 (4)	0–8.7	χ2 = 0.8997 df = 3 *P* = 0.63773	2 (4)	0–8.7	χ2 = 0.7991 df = 3 *P* = 0.670635	0	N.A	N.A
Kunduz	25	18 (72)	54.4–89.6		4 (16)	1.6–30.4		2 (8)	0–18.6		1 (4)	0–11.7		2 (8)	0–18.6	
Mazar-i-Sharif	11	7 (63.6)	35.2–92.1		2 (18)	0–41		1 (9)	0–26.1		0 (0)	0–0		0	N.A	
Takhar	10	8 (80)	55.2–100		2 (20)	0–44.8		0	N.A		1 (10)	0–28.6		0	N.A	
Keeping condition																
Stray	63	42 (66.7)	55–78.3	χ2 = 0.1391 df = 1 *P* = 0.709168	10 (15.9)	6.8–24.9	χ2 = 0.5219 df = 1 *P* = 0.470036	2 (3.2)	0–7.5	χ2 = 1.1944 df = 1 *P* = 0.274438	3 (4.8)	0–10	χ2 = 0.2574 df = 1 *P* = 0.611912	2 (3.2)	0–7.5	N.A
Privately owned	37	26 (70.3)	55.5–85		8 (21.6)	8.4–34.9		3 (8.1)	0–16.9		1 (2.7)	0–7.9		0	N.A	
Sex																
Female	51	31 (60.8)	47.4–74.2	χ2 = 6.492 df = 2 *P* = 0.038929	9 (17.6)	7.2–28.1	χ2 = 0.5849 df = 2 *P* = 0.746434	2 (3.9)	0–9.2	χ2 = 1.4006 df = 2 *P* = 0.496446	2 (3.9)	0–9.2	χ2 = 0.0395 df = 2 *P* = 0.842432	1 (2)	0–5.8	χ2 = 0.0193 df = 2 *P* = 0.889444
Male	42	34 (81)	69.1–92.8		7 (16.7)	5.4–27.9		2 (4.8)	0–11.2		2 (4.8)	0–11.2		1 (2.4)	0–7	
No data	7	3 (42.9)	6.2–79.5		2 (28.6)	0–62		1 (14.3)	0–40.2		0 (0)	0–0		0	N.A	
Age																
< 1 year	44	28 (63.6)	49.4–77.9	χ2 = 2.1842 df = 3 *P* = 0.335519	9 (20.5)	8.5–32.4	χ2 = 0.4543 df = 3 *P* = 0.796792	1 (2.3)	0–6.7	χ2 = 1.6407 df = 3 *P* = 0.200232	2 (4.5)	0–10.7	χ2 = 1.5575 df = 3 *P* = 0.458977	1 (2.3)	0–6.7	χ2 = 0.0003 df = 3 *P* = 0.987176
1–3 years	45	30 (66.7)	52.9–80.4		7 (15.6)	5.0–26.1		4 (8.9)	0.6–17.2		1 (2.2)	0–6.5		1 (2.2)	0–6.5	
3–5 years	9	8 (88.9)	68.4–100		2 (22.2)	0–49.4		0	N.A		1 (11.1)	0–31.6		0	N.A	
> 5 years	2	2 (100)	100–100		0	N.A		0	N.A		0 (0)	0–0		0	N.A	

*P* values were calculated with Chi-square test

Significant variables (*P* values < 0.05) are marked in bold

^a^Confidence interval

^b^Chi-square

^c^Degree of freedom

**Fig. 2:**
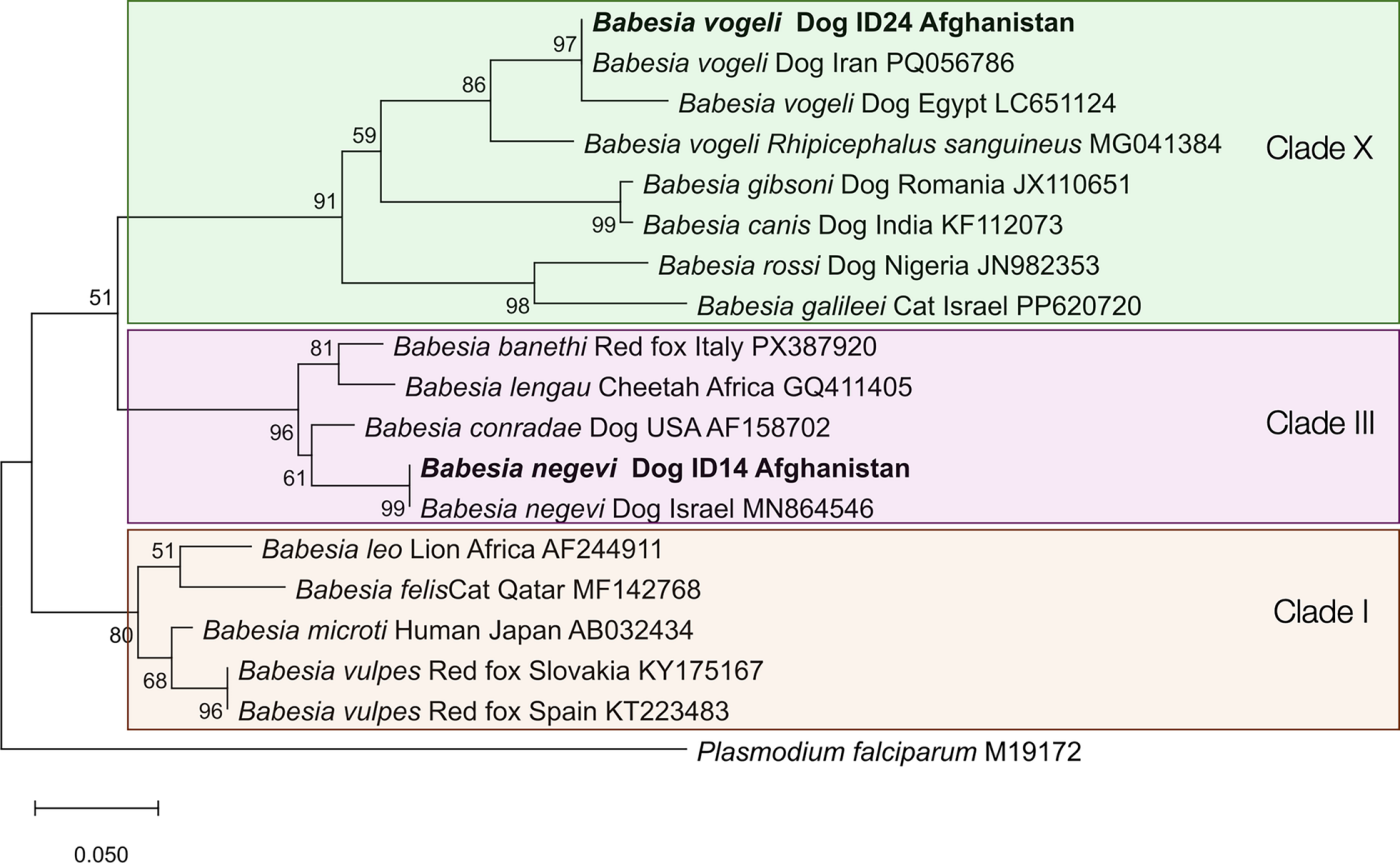
Phylogenetic tree of 18S rDNA gene sequences. *Babesia* sequences from the present study (in bold, *Babesia vogeli*: PZ279723; *Babesia negevi*: PZ276696) are compared with those of related taxa available in GenBank. Accession numbers, host, and country of origin are indicated, along with the main *Babesia* clusters (Clade I, Clade III, and Clade X) [53]. The tree was inferred using the Maximum Likelihood method under the Tamura (1992) model; the shown topology corresponds to the highest log likelihood (− 1238.13). Bootstrap values (8000 replicates) are reported at the nodes (values < 50 not shown). Evolutionary rate variation among sites was modeled using a discrete Gamma distribution (+ G, α = 0.2742). The final alignment included 240 positions after excluding sites with < 95% coverage. *Plasmodium falciparum* (M19172) was used as the outgroup

Overall, five dogs (5%; CI 0.7–9.3%) were infected with *Babesia* spp. (Table 3, Fig. 1). Of these, 4/5 were infected with *Babesia vogeli* (18S rDNA 100% nucleotide identity with a canine isolate from China, MK881090), and one dog from Mazar-i-Sharif was infected with *Babesia negevi* (18S rDNA 100% nucleotide identity with a canine isolate from Israel, MN864544). Accordingly, phylogenetic reconstruction placed the *B. vogeli* sequence obtained herein within Clade X (i.e., *Babesia* spp. sensu stricto), and the *B. negevi* isolate was grouped with other small *Babesia* spp. belonging to the Western Clade (i.e., Clade III) (Fig. 2).

**Fig. 3:**
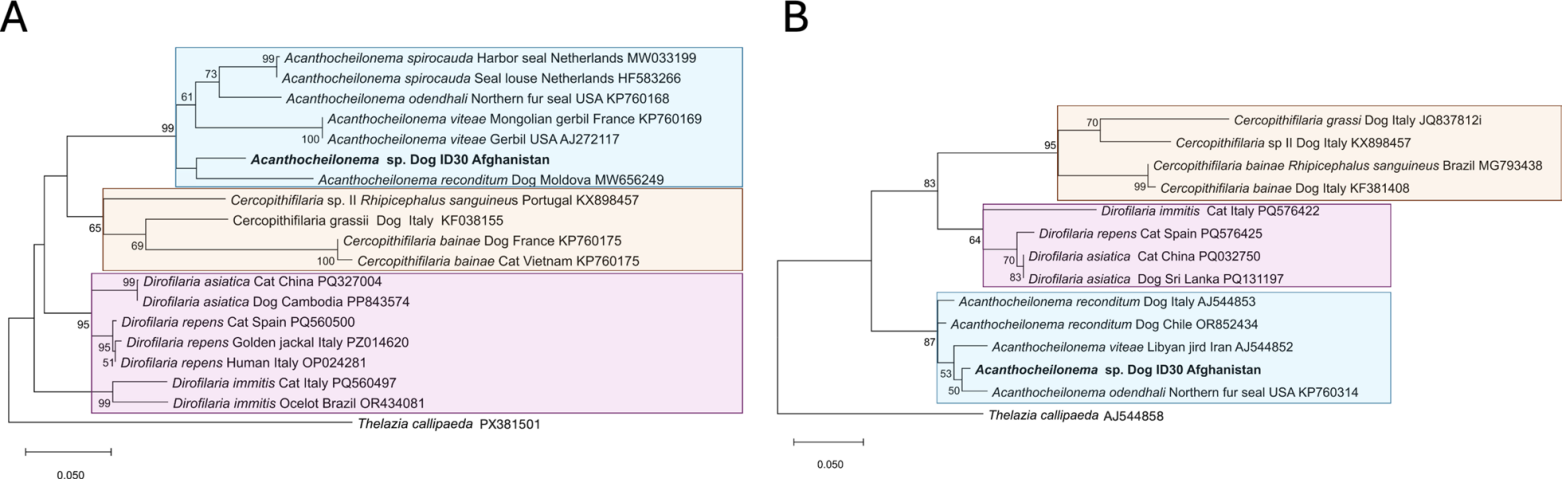
Phylogenetic trees of *Acanthocheilonema* sp. based on *cox*1 (**A**) and *12S* (**B**) gene sequences. Sequences from the present study (in bold, *12S*: PZ342259; *cox*1: PZ339976) are compared with those in GenBank; accession numbers, host, and country of origin are indicated. The *cox1* tree (**A**) was inferred under the Tamura (1992) model (log likelihood − 2860.28); the *12S* tree (**B**) was inferred under the Hasegawa–Kishino–Yano (1985) model (log likelihood − 863.95). Bootstrap values (2000 replicates) are shown at the nodes (values < 50 not shown). Rate variation among sites was modeled with a Gamma distribution (+ G, α = 1.1957) for *cox*1 and with a proportion of invariant sites (52.19%) for 12S. The final datasets comprised 648 positions (19 sequences) for *cox*1 and 228 positions (14 sequences) for 12S. *Thelazia callipaeda* was used as the outgroup (*cox*1: PX381501; *12S*: AJ544858)

*Acanthocheilonema* sp. DNA was amplified from four dog samples (i.e., two dogs from Kabul, one from Kunduz, and one from Takhar), and the resulting sequences were identical across both genetic markers (*cox*1 and *12S*) (Table 3, Fig. 1). However, sequencing of the *cox*1 and *12S* genes did not provide species-level identification (i.e., *cox*1: 92.1% nucleotide identity with the sequence of *A. reconditum*, JF461456; *12S rRNA*: 95.2% nucleotide identity with both the sequence of *A. reconditum*, PP214452, and *Acanthocheilonema viteae*, AP017679). Accordingly, interspecific pairwise comparison of the *cox*1 gene nucleotide sequences (207 bp) between *Acanthocheilonema* sp. (present study) and other congeners in GenBank (Table 4) showed the highest homology with *Acanthocheilonema dracunculoides* (i.e., 9.4% difference)*.* Consistently, phylogenetic analyses (Fig. 3a–*cox*1 gene; Fig. 3b–12S gene) placed the sequence of *Acanthocheilonema* sp. herein identified within the *Acanthocheilonema* spp. cluster but with low bootstrap values compared with other sequences of the same genus.

**Table 4:** Intra- and interspecific nucleotide variation percentages between *cox*1 nucleotide sequences (207 bp) of *Acanthocheilonema* sp. and other related sequences

	*Acanthocheilonema* sp.	*Acanthocheilonema dracunculoides*	*Acanthocheilonema reconditum*	*Acanthocheilonema viteae*	*Acanthocheilonema spirocauda*	*Dirofilaria immitis*	*Dirofilaria asiatica*	*Dirofilaria repens*
*Acanthocheilonema* sp.								
*Acanthocheilonema dracunculoides*	0.0949							
*Acanthocheilonema reconditum*	0.1126	0.1142						
*Acanthocheilonema viteae*	0.1359	0.1305	0.1571					
*Acanthocheilonema spirocauda*	0.1003	0.1019	0.1329	0.0945				
*Dirofilaria immitis*	0.1966	0.1910	0.1750	0.2012	0.1641			
*Dirofilaria asiatica*	0.1581	0.2002	0.1830	0.1572	0.1852	0.1370		
*Dirofilaria repens*	0.1511	0.1810	0.1639	0.1643	0.1435	0.1360	0.0394

**Fig. 4:**
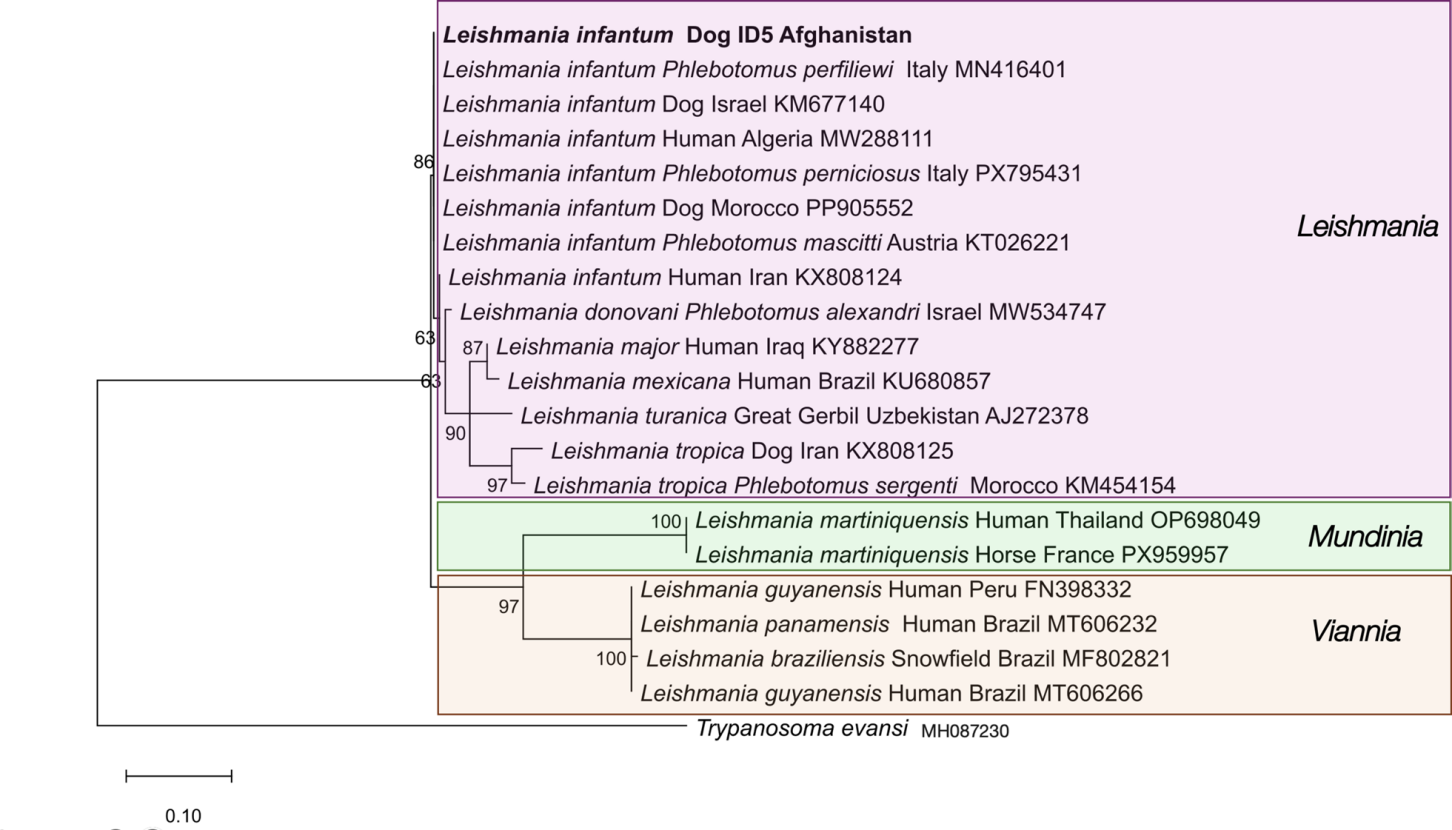
Phylogenetic tree of *Leishmania* spp. based on ITS1 region sequences. Sequences generated in this study (in bold, PZ269912) are compared with homologous sequences from GenBank; accession numbers, host species, and geographic origin are indicated, along with the main *Leishmania* clusters (*Leishmania*, *Mundinia*, *Viannia*) [54]. The tree was inferred using the Maximum Likelihood method under the Kimura 2-parameter model (log likelihood − 884.43). Bootstrap values (2000 replicates) are reported at the nodes (values < 50 not shown). The final dataset included 21 sequences and 181 positions after excluding sites with < 95% coverage. *Trypanosoma evansi* was used as the outgroup (MH087230)

Two dogs from Kunduz (8%, 95% CI 0–18.6%) were positive for *Leishmania* sp. kDNA by qPCR (Table 3, Fig. 1). One of these was sequenced by cPCR (ITS1 region) and matched the *L. infantum* isolate from a dog in Italy (AN JX945645). Sequencing of the other qPCR-positive sample using ITS1 and *Hsp70* PCR failed. Phylogenetic analyses clustered the sequence within the *Leishmania* clade, regardless of host species or geographical origin, and it was clearly distinct from the *Mundinia* and *Viannia* groups (Fig. 4).

No samples tested positive for *Ehrlichia* spp./*Anaplasma* spp. DNA.

Accession numbers for representative sequences of pathogens detected in this study were deposited in the GenBank database (*12S Acanthocheilonema* sp.: PZ342259; *cox*1 *Acanthocheilonema* sp.: PZ339976; *18S Hepatozoon canis*: PZ337661–PZ337662; *18S Babesia vogeli*: PZ279723; *18S Babesia negevi*: PZ276696; ITS1 *Leishmania infantum:* PZ269912).

## Discussion

The data presented provide the first epidemiological survey of CVBPs in Afghanistan, with *H. canis* the most prevalent, followed by *Bvb*, *Babesia* spp.*, Acanthocheilonema* sp., and *L. infantum*.

The high prevalence of *H. canis* (i.e., 68%) confirms its widespread circulation in dogs and aligns with previous reports of this infection in confined settings, such as Iran, Pakistan, and Kyrgyzstan [28, 29], suggesting a massive presence of the *Rhipicephalus sanguineus* sensu lato vectors in the absence of proper treatment and preventive measures [30, 31].

Similarly, *B. vogeli* was the most frequently detected piroplasm, confirming its global distribution, including in South-Central Asia [32]. As noted above, this species has already been detected in Iran, Pakistan, and Kyrgyzstan and is associated with *R. sanguineus* s.l. ticks [29, 33–35]. In addition to *B. vogeli*, other species of this genus have been documented in dogs from Western and Central Asia, including large piroplasms (i.e., *Babesia canis* in Iran) [36] and small ones (i.e., *Babesia gibsoni* in Pakistan and *Babesia vulpes* in Kyrgyzstan) [33, 37], underscoring the importance of blood smear examination, which remains the most feasible approach for diagnosing clinical babesiosis in these settings. Specifically, accurate differentiation between large and small *Babesia* spp., achievable through blood cytology, is crucial from a clinical perspective, as large *Babesia* species are less pathogenic than small forms and the latter require more complex therapeutic approaches than imidocarb treatment [18]. The detection of *B. negevi* was unprecedented, as it had previously been reported only in dogs from Israel, the Palestinian Authority, and Jordan [38, 39], suggesting a wider distribution than currently recognized.

In this context, dog relocation events, such as those that occurred during the 2021 evacuation from Afghanistan following the return of the Taliban regime [40] may have contributed to the spread of infected dogs into non-endemic areas and facilitated the establishment of *Babesia* spp., due to the presence of competent tick vectors [41].

The zoonotic *Bvb*, with 14 confirmed cases, was the second most prevalent pathogen detected in this study, consistent with previous reports in dogs from neighboring Iran [42, 43], where other zoonotic *Bartonella* species (i.e., *B. clarridgeiae*, *B. rochalimae*, and *B. henselae*) were also identified [42–45].

The detection of *Leishmania* DNA in two dogs from Kunduz, with ITS1 sequencing supporting *L. infantum* identification in one sample, provides the first molecular evidence of its occurrence in canine hosts in Afghanistan, where *Leishmania* spp. amastigotes were observed in the skin cytology of sick dogs [22]. In addition, the *Leishmania donovani* complex was previously diagnosed in autochthonous human cases of visceral leishmaniasis [46], and *L. infantum* was detected in military personnel deployed in Afghanistan [47] and in human patients from Uzbekistan and Tajikistan [48]. Overall, data on leishmaniasis in Afghanistan are currently fragmented but suggest high endemicity and should be corroborated by entomological surveys and serological screening of vertebrate hosts to provide a clearer epidemiological picture of the infection in both human and animal settings. The failure to sequence the remaining qPCR-positive sample was likely related to the lower sensitivity of cPCR compared to qPCR, and *Hsp70* amplification may have been further limited by the lower copy number of this target compared with ITS1. Finally, although ITS1 sequencing supported the identification of *L. infantum* in one dog, additional species-informative markers, such as cysteine protease B (*cpb*), may further strengthen discrimination within the *L. donovani* complex [49, 50].

The finding of a sequence type most likely corresponding to a single *Acanthocheilonema* sp. in dogs from three distinct regions of Afghanistan may indicate that these filarioid infections, as well as their arthropod vectors, are present across the country. However, sequence analyses and pairwise comparisons based on the *cox*1 and *12S* genes did not yield a species-level identification, as further confirmed by the genetic distances observed in both phylogenetic reconstructions. Therefore, these data suggest that the detected *Acanthocheilonema* sp. sequence could represent a new taxon of the genus, though the low molecular resolution of such short mitochondrial fragments, as well as the absence of morphological data, prevent a definitive conclusion. Although *Acanthocheilonema* spp. are generally regarded as having low clinical relevance in dogs [51], their detection warrants further investigation of canine filarioids in Afghanistan, including blood examination by Knott’s test to morphologically characterize microfilariae and clarify the taxonomic status of these sequence types.

The authors acknowledge that a limitation of this study is the absence of hematological and biochemical analyses, which are needed for a comprehensive clinical evaluation and for assessing potential associations between CVBPs and clinical findings. These analyses would have been particularly valuable for evaluating the clinical impact of the co-infections identified in this study (e.g., *L. infantum* and *Bartonella* spp.). In addition, using dried blood spots as the sole biological matrix may have reduced sensitivity for pathogen detection compared with more appropriate samples (e.g., lymph node aspirates for *Leishmania* spp., buffy coat for *Hepatozoon* and *Babesia* spp., and whole blood for circulating microfilariae). However, this approach was driven by field conditions, as dried blood spots are a low-cost, easily transportable sampling method [52] and the only option in settings characterized by logistical, socioeconomic, and political constraints, such as Afghanistan.

## Conclusions

To the best of our knowledge, this is the first epidemiological study on CVBPs in Afghanistan, highlighting the circulation of *H*. *canis*, *Bvb*, *L. infantum*, *B. vogeli*, *B. negevi*, and *Acanthocheilonema* sp. in the examined canine population. Overall, the data underscore the importance of implementing ectoparasite control measures for owned dogs and the need to control feral animal populations through spay-and-neuter campaigns to mitigate transmission risk to both animal and human populations.

## Data Availability

All data supporting the main conclusions of this study are included in the manuscript.

## Abbreviations

CVBD: Canine vector-borne disease
CVBP: Canine vector-borne pathogen
*Bvb*: *Bartonella vinsonii* subspecies *berkhoffii*
EDTA: Ethylenediaminetetraacetic acid
cPCR: Conventional polymerase chain reaction
qPCR: Quantitative polymerase chain reaction
UV: Ultraviolet
DNA: Deoxyribonucleic acid
kDNA: Kinetoplast DNA
rDNA: Ribosomal DNA
ITS: Internal transcribed spacer
*cox*1: Cytochrome c oxidase subunit I
AIC: Akaike Information Criterion
MAFFT: Multiple Alignment using Fast Fourier Transform
CIPRES: Cyber-Infrastructure for Phylogenetic Research
AN: Accession number
CI: Confidence intervals
df: Degree of freedom
χ^2^: Chi-square
ML: Maximum likelihood
Hsp70: 70 Kilodalton heat shock proteins
Cpb: Cysteine protease B

## References

[1] Rojas A, Dantas-Torres F, Mendoza-Roldan JA, Otranto D. Emergent, re-emerging, or just ignored: the many lives of cat and dog parasites. Trends Parasitol. 2026. 10.1016/j.pt.2026.05.007. 42225488 10.1016/j.pt.2026.05.007

[2] de Souza WM, Weaver SC. Effects of climate change and human activities on vector-borne diseases. Nat Rev Microbiol. 2024;22:476–91. 10.1038/s41579-024-01026-0. 38486116 10.1038/s41579-024-01026-0

[3] Nguyen V, Colella V, Greco G, Fang F, Nurcahyo W, Hadi U, et al. Molecular detection of pathogens in ticks and fleas collected from companion dogs and cats in East and Southeast Asia. Parasit Vectors. 2020;13:420. 10.1186/s13071-020-04288-8. 32799914 10.1186/s13071-020-04288-8 PMC7429691

[4] Otranto D, Dantas-Torres F, Breitschwerdt EB. Managing canine vector-borne diseases of zoonotic concern: part one. Trends Parasitol. 2009;25:157–63. 19269898 10.1016/j.pt.2009.01.003

[5] Chomel BB, Boulouis H-J, Maruyama S, Breitschwerdt EB. *Bartonella* spp. in pets and effect on human health. Emerg Infect Dis. 2006;12:389–94. 10.3201/eid1203.050931. 16704774 10.3201/eid1203.050931 PMC3291446

[6] Zarea AAK, Tempesta M, Odigie AE, Mrenoshki D, Fanelli A, Martella V, et al. The global molecular prevalence of *Bartonella* spp. in cats and dogs: a systematic review and meta-analysis. Transbound Emerg Dis. 2023;2023:7867562. 10.1155/2023/7867562. 40303778 10.1155/2023/7867562 PMC12017235

[7] Marié J-L, Fournier P-E, Rolain J-M, Briolant S, Davoust B, Raoult D. Molecular detection of *Bartonella quintana, B. elizabethae, B. koehlerae, B. doshiae, B. taylorii, *and* Rickettsia felis* in rodent fleas collected in Kabul Afghanistan. Am J Trop Med Hyg. 2006;74:436–9. 16525103

[8] Dantas-Torres F, Solano-Gallego L, Baneth G, Ribeiro VM, de Paiva-Cavalcanti M, Otranto D. Canine leishmaniosis in the Old and New Worlds: unveiled similarities and differences. Trends Parasitol. 2012;28:531–8. 22995719 10.1016/j.pt.2012.08.007

[9] Saridomichelakis MN, Baneth G, Colombo S, Dantas-Torres F, Ferrer L, Fondati A, et al. World Association for Veterinary Dermatology consensus statement for diagnosis, and evidence-based clinical practice guidelines for treatment and prevention of canine leishmaniosis. Vet Dermatol. 2025;36:723–87. 10.1111/vde.70006. 40745695 10.1111/vde.70006 PMC12590123

[10] Manathunga T, Carbonara M, Nekouei O, Mendoza-Roldan JA, Tam WYJ, Beugnet F, et al. High prevalence of vector-borne pathogens in the blood of clinically healthy dogs in Hong Kong. Parasites Vectors. 2025;18:289. 10.1186/s13071-025-06853-5. 40685364 10.1186/s13071-025-06853-5 PMC12278506

[11] Hosseini SH, Manshori-Ghaishghorshagh F, Ramezani M, Nayebzadeh H, Ahoo MB, Eslamian A, et al. Canine microfilaraemia in some regions of Iran. Parasit Vectors. 2022;15:90. 10.1186/s13071-022-05209-7. 35303931 10.1186/s13071-022-05209-7 PMC8932200

[12] Dantas-Torres F, de Sousa-Paula LC, Otranto D. The *Rhipicephalus sanguineus* group: updated list of species, geographical distribution, and vector competence. Parasit Vectors. 2024;17:540. 10.1186/s13071-024-06572-3. 39731169 10.1186/s13071-024-06572-3 PMC11681662

[13] Schnittger L, Ganzinelli S, Bhoora R, Omondi D, Nijhof AM, Florin-Christensen M. The Piroplasmida *Babesia*, *Cytauxzoon*, and *Theileria* in farm and companion animals: species compilation, molecular phylogeny, and evolutionary insights. Parasitol Res. 2022;121:1207–45. 10.1007/s00436-022-07424-8. 35098377 10.1007/s00436-022-07424-8

[14] Akhtardanesh B, Arfaee F, Zia-Addini A, Mostafavi S-M, Jajarmi M, Sazmand A. Molecular detection of *Hepatozoon canis* (Apicomplexa: Hepatozoidae) in ticks (Ixodida) collected from dogs in Kerman Iran. Persian J Acarol. 2025;14:1–10. 10.22073/pja.v14i1.85646.

[15] Otranto D, Mendoza-Roldan JA, Beugnet F, Baneth G, Dantas-Torres F. New paradigms in the prevention of canine vector-borne diseases. Trends Parasitol. 2024;40:500–10. 10.1016/j.pt.2024.04.009. 38744542 10.1016/j.pt.2024.04.009

[16] Dubey J, Alić A, Hodžić A, Lopez-Flores J, Baneth G. Hepatozoon infections in domestic and wild Carnivora: etiology, prevalence, clinical disease, diagnosis and treatment, and redescription of *Hepatozoon silvestris*, *H. martis*, and *H. ursi*. Parasit Vectors. 2025;18:391. 10.1186/s13071-025-06977-8. 40993772 10.1186/s13071-025-06977-8 PMC12462345

[17] Leisewitz A, Mrljak V, Dear J, Birkenheuer A. The diverse pathogenicity of various *Babesia* parasite species that infect dogs. Pathogens. 2023;12:1437. 10.3390/pathogens12121437. 38133320 10.3390/pathogens12121437 PMC10746086

[18] Solano-Gallego L, Sainz Á, Roura X, Estrada-Peña A, Miró G. A review of canine babesiosis: the European perspective. Parasit Vectors. 2016;9:336–336. 10.1186/s13071-016-1596-0. 27289223 10.1186/s13071-016-1596-0 PMC4902949

[19] Mosawi SH, Zarei Z, Shams M, Mohammadi K, Sajjadi SA. Environmental health and leishmaniasis by indication on Afghanistan: A review. Nriagu, J Ed Encyclopedia of Environmental Health, 2nd Edition. Amsterdam, Netherlands: Elsevier; 2019. p. 458–65. 10.1016/B978-0-12-409548-9.11740-3

[20] Otranto D, Dantas-Torres F, Mihalca AD, Traub RJ, Lappin M, Baneth G. Zoonotic parasites of sheltered and stray dogs in the era of the global economic and political crisis. Trends Parasitol. 2017;33:813–25. 10.1016/j.pt.2017.05.013. 28648798 10.1016/j.pt.2017.05.013

[21] Le Riche PD, Soe AK, Alemzada Q, Sharifi L. Parasites of dogs in Kabul. Afghanistan Br Vet J. 1988;144:370–3. 10.1016/0007-1935(88)90067-X. 3167551 10.1016/0007-1935(88)90067-X

[22] Nadim A, Rostami G. Epidemiology of cutaneous leishmaniasis in Kabul Afghanistan. Bull World Health Organ. 1974;51:45–9. 4549041 PMC2366244

[23] Tamura K. Estimation of the number of nucleotide substitutions when there are strong transition-transversion and G+C-content biases. Mol Biol Evol. 1992;9:678–87. 10.1093/oxfordjournals.molbev.a040752. 1630306 10.1093/oxfordjournals.molbev.a040752

[24] Hasegawa M, Kishino H, Yano T. Dating of the human-ape splitting by a molecular clock of mitochondrial DNA. J Mol Evol. 1985;22:160–74. 3934395 10.1007/BF02101694

[25] Kimura M. A simple method for estimating evolutionary rates of base substitutions through comparative studies of nucleotide sequences. J Mol Evol. 1980;16:111–20. 7463489 10.1007/BF01731581

[26] Kumar S, Stecher G, Suleski M, Sanderford M, Sharma S, Tamura K. MEGA12: molecular evolutionary genetic analysis version 12 for adaptive and green computing. Mol Biol Evol. 2024;41:msae63. 10.1093/molbev/msae263. 10.1093/molbev/msae263 PMC11683415 39708372

[27] Tamura K, Nei M, Kumar S. Prospects for inferring very large phylogenies by using the neighbor-joining method. PNAS. 2004;101:11030–5. 10.1073/pnas.0404206101. 15258291 10.1073/pnas.0404206101 PMC491989

[28] Altay K, Aydın M, Aytmirzakizi A, Jumakanova Z, Cunusova A, Dumanlı N. Molecular survey of hepatozoonosis in natural infected dogs: First detection and molecular characterisation of *Hepatozoon canis* in Kyrgyzstan. Kafkas Univ Vet Fak Derg. 2019;25:77–81. 10.9775/kvfd.2018.20352.

[29] Iatta R, Sazmand A, Nguyen V-L, Nemati F, Bahiraei Z, Mazhar Ayaz M, et al. Vector-borne pathogens in dogs of different regions of Iran and Pakistan. Parasitol Res. 2021;120:4219–28. 10.1007/s00436-020-06992-x. 33506332 10.1007/s00436-020-06992-x PMC8599219

[30] Baneth G, Allen K. Hepatozoonosis of dogs and cats. Vet Clin North Am Small Anim Pract. 2022;52:1341–58. 10.1016/j.cvsm.2022.06.011. 36336424 10.1016/j.cvsm.2022.06.011

[31] Schotte U, Binder A, Goller KV, Faulde M, Ruhl S, Sauer S, et al. Field survey and molecular characterization of apicomplexan parasites in small mammals from military camps in Afghanistan. Parasitol Res. 2023;122:1199–211. 10.1007/s00436-023-07820-8. 36944808 10.1007/s00436-023-07820-8 PMC10097762

[32] Fu B-K, Tang T, Yue M, Chen J-J, Su H, Huang X-B, et al. Mapping the global distribution of *Babesia* infections. Transbound Emerg Dis. 2025;2025:5889219. 10.1155/tbed/5889219. 41333613 10.1155/tbed/5889219 PMC12668841

[33] Altay K, Erol U, Sahin OF, Aydin MF, Aytmirzakizi A, Dumanli N. First molecular evidence of *Babesia vogeli, Babesia vulpes*, and *Theileria ovis* in dogs from Kyrgyzstan. Pathogens. 2023;12:1046. 10.3390/pathogens12081046. 37624006 10.3390/pathogens12081046 PMC10460036

[34] Saghir AA, Imran R, Kamran A, Wasim S, Matiullah K, Kashif H. et al. Molecular occurrence of canine babesiosis in rural dog population in Pakistan. Trop Biomed. 2018;35:593–603. 33601746

[35] Tayyub M, Ashraf K, Lateef M, Anjum AA, Ali MA, Ahmad N, et al. Genetic diversity of canine *Babesia* species prevalent in pet dogs of Punjab. Pakistan Animals. 2019;9:439. 10.3390/ani9070439. 31337004 10.3390/ani9070439 PMC6680441

[36] Khanmohammadi M, Zolfaghari-Emameh R, Arshadi M, Razmjou E, Karimi P. Molecular identification and genotyping of *Babesia canis* in dogs from Meshkin Shahr County, Northwestern Iran. J Arthropod Borne Dis. 2021;15:97–107. 10.18502/jad.v15i1.6489. 34277859 10.18502/jad.v15i1.6489 PMC8271237

[37] Ali M, Rasool M, Ali A, Muqaddas H, Naeem M, Farooq M, et al. Molecular prevalence, epidemiology, and phylogenetic analysis of *Babesia microti* in dogs with a note on its impact on host hematological profile. Vet Parasitol Reg Stud Reports. 2024;55:101114. 10.1016/j.vprsr.2024.101114. 39326966 10.1016/j.vprsr.2024.101114

[38] Baneth G, Nachum-Biala Y, Birkenheuer AJ, Schreeg ME, Prince H, Florin-Christensen M, et al. A new piroplasmid species infecting dogs: morphological and molecular characterization and pathogeny of *Babesia negevi* n sp. Parasit Vectors. 2020;13:130. 10.1186/s13071-020-3995-5. 32312309 10.1186/s13071-020-3995-5 PMC7171826

[39] Far D, Takács N, Gyurkovszky M, Solymosi N, Farkas R. Ticks and tick-borne infections of dogs in two Jordanian shelters. Vector Borne Zoonotic Dis. 2021;21:573–8. 10.1089/vbz.2021.0026. 34077691 10.1089/vbz.2021.0026

[40] Jackson S. Afghanistan: Pen Farthing “on his way home with his dogs and cats” after charter plane picks them up from Kabul. Sky News. 2021 Aug 28; https://news.sky.com/story/afghanistan-charter-plane-arrives-in-kabul-to-collect-pen-farthing-and-his-animals-12393262. Accessed 14 May 2026.

[41] Simmons KE, Hoffman CL. Dogs on the move: factors impacting animal shelter and rescue organizations’ decisions to accept dogs from distant locations. Animals. 2016;6:11. 10.3390/ani6020011. 26848694 10.3390/ani6020011 PMC4773738

[42] Greco G, Sazmand A, Goudarztalejerdi A, Zolhavarieh SM, Decaro N, Lapsley WD, et al. High prevalence of *Bartonella* sp. in dogs from Hamadan. Iran Am J Trop Med Hyg. 2019;101:749–52. 10.4269/ajtmh.19-0345. 31436150 10.4269/ajtmh.19-0345 PMC6779217

[43] Shamshiri Z, Goudarztalejerdi A, Zolhavarieh SM, Kamalpour M, Sazmand A. Molecular Identification of *Bartonella* species in dogs and arthropod vectors in Hamedan and Kermanshah, Iran. Iran Vet J. 2023;19:104–16. 10.22055/IVJ.2022.325115.2436.

[44] Alempour Rajabi S, Ownagh A, Hadian M. Molecular survey and phylogenetic analysis of *Bartonella henselae* and *Bartonella clarridgeiae* in dogs from northwest Iran. J Zoonotic Dis. 2024;8:610–20. 10.22034/jzd.2024.18137.

[45] Samsami S, Ghaemi M, Sharifiyazdi H. Molecular detection and phylogenetic analysis of ‘*Candidatus* Bartonella merieuxii’in dogs and its effect on hematologic parameters. Comp Immunol Microbiol Infect Dis. 2020;72:101504. 10.1016/j.cimid.2020.101504. 32544730 10.1016/j.cimid.2020.101504

[46] Leslie T, Saleheen S, Sami M, Mayan I, Mahboob N, Fiekert K, et al. Visceral leishmaniasis in Afghanistan. Can Med Assoc J. 2006;175:245. 10.1503/cmaj.060202. 16880443 10.1503/cmaj.060202 PMC1513431

[47] CDC. Two cases of visceral leishmaniasis in U.S. military personnel--Afghanistan, 2002–2004. MMWR Morb Mortal Wkly Rep. 2004;53:265–8. 15057193

[48] Alam MZ, Kovalenko DA, Kuhls K, Nasyrova RM, Ponomareva VI, Fatullaeva AA, et al. Identification of the agent causing visceral leishmaniasis in Uzbeki and Tajiki foci by analysing parasite DNA extracted from patients’ Giemsa-stained tissue preparations. Parasitology. 2009;136:981–6. 10.1017/S0031182009006465. 19549349 10.1017/S0031182009006465

[49] Van der Auwera G, Ravel C, Verweij JJ, Bart A, Schönian G, Felger I. Evaluation of four single-locus markers for *Leishmania* species discrimination by sequencing. J Clin Microbiol. 2014;52:1098–104. 10.1128/JCM.02936-13. 24452158 10.1128/JCM.02936-13 PMC3993476

[50] Hide M, Bañuls A-L. Species-specific PCR assay for *L. infantum/L. donovani* discrimination. Acta Trop. 2006;100:241–5. 10.1016/j.actatropica.2006.10.012. 17141723 10.1016/j.actatropica.2006.10.012

[51] Otranto D, Dantas-Torres F, Brianti E, Traversa D, Petrić D, Genchi C, et al. Vector-borne helminths of dogs and humans in Europe. Parasit Vectors. 2013;6:16. 10.1186/1756-3305-6-16. 23324440 10.1186/1756-3305-6-16 PMC3564894

[52] Rojas A, Germitsch N, Oren S, Sazmand A, Deak G. Wildlife parasitology: sample collection and processing, diagnostic constraints, and methodological challenges in terrestrial carnivores. Parasit Vectors. 2024;17:127. 10.1186/s13071-024-06226-4. 38481271 10.1186/s13071-024-06226-4 PMC10938792

[53] Jalovecka M, Sojka D, Ascencio M, Schnittger L. *Babesia* life cycle - when phylogeny meets biology. Trends Parasitol. 2019;35:356–68. 10.1016/j.pt.2019.01.007. 30733093 10.1016/j.pt.2019.01.007

[54] Rojas A, Mendoza-Roldan JA, Alfaro-Segura P, Solano-Barquero A, Otranto D. Partners in time: cospeciation and host-switching shape the evolution of *Leishmania* parasites. PLoS Negl Trop Dis. 2025;19:e0013814. 10.1371/journal.pntd.0013814. 41433316 10.1371/journal.pntd.0013814 PMC12725659

[55] Gubbels J, De Vos A, Van der Weide M, Viseras J, Schouls L, De Vries E, et al. Simultaneous detection of bovine *Theileria* and *Babesia* species by reverse line blot hybridization. J Clin Microbiol. 1999;37:1782–9. 10325324 10.1128/jcm.37.6.1782-1789.1999 PMC84950

[56] Casiraghi M, Anderson TJ, Bandi C, Bazzocchi C, Genchi C. A phylogenetic analysis of filarial nematodes: comparison with the phylogeny of *Wolbachia* endosymbionts. Parasitology. 2001;122:93–103. 10.1017/s0031182000007149. 11197770 10.1017/s0031182000007149

[57] Otranto D, Brianti E, Latrofa MS, Annoscia G, Weigl S, Lia RP, et al. On a *Cercopithifilaria* sp. transmitted by *Rhipicephalus sanguineus*: a neglected, but widespread filarioid of dogs. Parasit Vectors. 2012;5:1. 10.1186/1756-3305-5-1. 22212459 10.1186/1756-3305-5-1 PMC3259067

[58] Gasser RB, Chilton NB, Hoste H, Beveridge I. Rapid sequencing of rDNA from single worms and eggs of parasitic helminths. Nucleic Acids Res. 1993;21:2525–6. 10.1093/nar/21.10.2525. 8506152 10.1093/nar/21.10.2525 PMC309567

[59] Parola P, Roux V, Camicas J-L, Baradji I, Brouqui P, Raoult D. Detection of ehrlichiae in African ticks by polymerase chain reaction. Trans R Soc Trop Med Hyg. 2000;94:707–8. 10.1016/S0035-9203(00)90243-8. 11198664 10.1016/s0035-9203(00)90243-8

[60] Francino O, Altet L, Sanchez-Robert E, Rodriguez A, Solano-Gallego L, Alberola J, et al. Advantages of real-time PCR assay for diagnosis and monitoring of canine leishmaniosis. Vet Parasitol. 2006;137:214–21. 10.1016/j.vetpar.2006.01.011. 16473467 10.1016/j.vetpar.2006.01.011

[61] El Tai NO, El Fari M, Mauricio I, Miles MA, Oskam L, El Safi SH, et al. *Leishmania donovani*: intraspecific polymorphisms of Sudanese isolates revealed by PCR-based analyses and DNA sequencing. Exp Parasitol. 2001;97:35–44. 10.1006/expr.2001.4592. 11207112 10.1006/expr.2001.4592

[62] Van der Auwera G, Maes I, De Doncker S, Ravel C, Cnops L, Van Esbroeck M, et al. Heat-shock protein 70 gene sequencing for *Leishmania* species typing in European tropical infectious disease clinics. Euro Surveill. 2013;18:20543. 10.2807/1560-7917.es2013.18.30.20543. 23929181 10.2807/1560-7917.es2013.18.30.20543

[63] Diniz PPVDP, Maggi RG, Schwartz DS, Cadenas MB, Bradley JM, Hegarty B, et al. Canine bartonellosis: serological and molecular prevalence in Brazil and evidence of co-infection with *Bartonella henselae* and *Bartonella vinsonii* subsp *berkhoffii*. Vet Res. 2007;38:697–710. 10.1051/vetres:2007023. 17583666 10.1051/vetres:2007023

